# Differential and Linear properties of vectorial boolean functions based on chi

**DOI:** 10.1007/s12095-023-00639-1

**Published:** 2023-04-26

**Authors:** Silvia Mella, Alireza Mehrdad, Joan Daemen

**Affiliations:** Digital Security Group, Radboud University, Nijmegen, The Netherlands

**Keywords:** Chi Mapping, Differential Cryptan0. alysis, Differential Probability, Linear Cryptanalysis, Linear Approximation, Correlation, 94A60, 06E30

## Abstract

To evaluate the security of a cryptographic primitive, investigating its resistance against differential and linear cryptanalysis is required. Many modern cryptographic primitives repeatedly apply similar round functions alternated with the addition of round keys or constants. A round function usually consists of a non-linear mapping and a number of linear mappings. The non-linear mapping $\chi$ is used in different cryptographic primitives such as Keccak and Subterranean. An alternative version of $\chi$ is used in Ascon and the non-linear layer of Simon has the same differential and linear properties of $\chi$. The mapping $\chi$ can be applied to strings with different lengths. For instance, it can be parallelly applied to small-length strings as in Keccak, where it works on 5-bit strings, or it can be applied to big-length strings as in Subterranean, where it works on a string of length 257. Investigating the differential and linear properties of $\chi$ working on alternative lengths of strings, provides useful information to designers to make a better choice for the non-linear layer. Some differential properties of $\chi$ have been analyzed in [8] and in this work we provide a revised presentation of them. We then extend this study and we analyze linear propagation properties of $\chi$. Thanks to these additional results, we extend the comparison between the application of parallel instances of $\chi$ on small-length strings and the application of a single instance of $\chi$ on a big-length string. We show how we can apply the results of this study also to the non-linear layers of Ascon and Simon thanks to their affine-equivalence with $\chi$.

## Introduction

Modern ciphers and cryptographic permutations consist of the repeated application of a round function alternated with the addition of round keys or constants. A round function can usually be divided into a non-linear layer and a linear layer. The non-linear layer provides *confusion*, while the linear layer mixes different parts of the state and provides *diffusion*.

The design of modern cryptographic primitives like AES [10] and Keccak-$f$ permutation [2] is motivated by the *wide trail strategy*, that underlies the design of round functions with good resistance against differential and linear cryptanalysis. In a nutshell, it requires the absence of high-probability differential propagation patterns, called *differential trails*, and high-correlation propagation patterns, called *linear trails*.

When analyzing differential and linear trails, the differential and linear propagation properties of the non-linear layer play an important role. In particular, one is interested in the probability of the propagation of a difference $b$ at the input of the non-linear layer to a difference $a$ at its output. The same holds for linear cryptanalysis where one exploits the probability of the propagation of an input mask $u$ to an output mask $v$. An ordered pair of input-output differences $(b, a)$ is called a *differential* and an ordered pair of input-output masks $(u, v)$ is called a *linear approximation*.

For a non-linear map $f:\mathbb {F}_2^n \rightarrow \mathbb {F}_2^n$ there exist $2^{2n}$ differentials and $2^{2n}$ linear approximations. The number of input pairs satisfying each differential and linear approximation are usually arranged in a $2^n$ by $2^n$ array, called the *difference distribution table* ($\textrm{DDT}$) and linear approximation table ($\textrm{LAT}$), respectively. For small domains the $\textrm{DDT}$ and $\textrm{LAT}$ have manageable sizes so one can build them by brute-force, but it is not the case when the map works on a large dimension.

Keccak-$f$ and some other cryptographic primitives, like Xoodoo [6] and Subterranean [7], make use of the non-linear mapping $\chi$. In the case of $\chi$, one can compute the probability of a differential or a linear approximation, without forming the $\textrm{DDT}$ and $\textrm{LAT}$ but using the method introduced in [5]. It means that even if $\chi$ works on a big-length string, computing the probability of a differential or linear approximation is still practical.

In addition to the computation of the probability of differentials and linear approximations, determining the number of differentials and linear approximations with a given probability gives useful information about how a non-linear layer may perform in the wide trail strategy: fewer differentials (resp. linear approximations) with high probability results in less opportunity to form differential (resp. linear) trails with high probability over multiple rounds. Therefore, when designing a cipher, it is useful to have the histograms with the number of high probability differentials and linear approximations for the alternative choices of the non-linear layer. While one can easily generate this histogram for the case where $\chi$ is applied to small-length strings in parallel, it is challenging when $\chi$ is applied to a big-length string.

In [8], we presented a method to compute the number of differentials over $\chi$ with a given probability and for arbitrary string lengths. In this work, we complement these results with an analysis of the linear propagation properties through $\chi$ and we investigate the differential properties of $\chi$ given a specific output difference and the linear properties of $\chi$ given a specific input mask. For the sake of completeness, we include in this work a revised explanation of the method and results presented in [8], since we will use them to compare the differential and linear propagation properties of $\chi$.

### Our contribution

In this work, we first present a method to compute the number of linear approximations over $\chi$ with a given correlation and for arbitrary string lengths. Using this method and the method in [8], we provide a comparison between the distribution of differentials and linear approximations in some well known permutations whose non-linear layer is based on $\chi$. For the permutations based on the parallel application of $\chi$ on short strings, we provide a comparison with $\chi$ applied on a single string whose length is the state size. Then, we provide a comparison between the number of 2-round differential and linear trail cores in Ascon and in Xoodoo and discuss how the results on differentials and linear approximations reflect also on 2-round trail cores. Finally, we study the propagation of 1-bit output differences to input differences and the propagation of 1-bit input masks to output masks. We discuss how this study can be applied to the non-linear layers of Simon [1] and Ascon [12], due to their similarity with the mapping $\chi$.

### Organization of the paper

In Section , we first provide our terminology and recall the specification of the mapping $\chi$ and the non-linear layers of Simon and Ascon. We then recall the main concepts of differential and linear cryptanalysis and explain why we can examine the properties of the non-linear layers of Simon and Ascon in the same way as we do for $\chi$. In Section , we give a revised explanation of the results presented in [8] on how to compute distribution of differentials over $\chi$. In Section , we investigate the distribution of linear approximations over $\chi$. In Section , we first present a comparison between differential and linear properties of $\chi$ and the number of 2-round differential and linear trail cores. Then we investigate the propagation of 1-bit output differences and input masks. Finally, in Section , we provide some final remarks.

## Preliminaries

In this section, we first briefly introduce the terminologies that we use in this paper. Then, we recall the specification of the non-linear mapping $\chi$, the round function and non-linear layer of Simon, and the non-linear layer of Ascon.

### Terminology

Let *s* be a binary string, we denote its *i*-th bit by $s_i$, where we start indexing from 0. Therefore, $i \in \{0, 1, \ldots , |s|-1\}$ when $|s|$ denotes the length of *s*.

Indexing of bits in a string outside the range $[0,|s|-1]$ is allowed by reducing the index modulo $|s|$. This allows us to consider circular strings, that means the first bit $s_0$ and last bit $s_{|s|-1}$ are neighbors. We call such strings *circles*. A string $s^\prime$ is a *sub-string* of circular string *s* if $|s^\prime | \le |s|$ and $\exists i \in [0, |s|-1]$ such that $s^\prime _j = s_{j+i}$ for all $j \in [0,|s^\prime | - 1]$. As an example, we say $s^\prime = 100011$ is a sub-string of circular string $s = 0111100$ since $s^\prime _j = s_{j+4}$ for all $j \in [0,5]$.

We use $s \lll t$ to indicate the circular rotation of a string *s* that moves bit in position *i* to position $i+t$. This notation is commonly used when *s* is seen as a CPU word where the bit at index 0 is the right-most bit. Notice that in our figures, we depict bits as disposed on a Cartesian axis with the origin on the left. Therefore, in our figures bit 0 is on the left, unless otherwise specified.

We write $s \Vert s^{\prime }$ for the concatenation of two strings *s* and $s^{\prime }$, and we use $(s)^m$ for the concatenation of *m* copies of *s*: $s\Vert s \Vert \ldots \Vert s$. Hence, the notation $(1)^{m}$ represents an all-one string of length *m* that we call a 1*-run*. A 1-run in a string should be preceded and followed by a 0-bit, except when there is no zero in the string. In this case, we say that the string contains a single 1-run.

Let $s_i \in \mathbb {F}_2$ be a bit of a string *s*, then we say $s_i$ is an *active* bit if $s_i = 1$, and is a *passive* bit otherwise. Then, the *Hamming weight* of a string $s \in \mathbb {F}_2^{n}$, denoted by $h$, is the number of active bits in *s*.

### Specification of $\chi$

The mapping $\chi$ is used as non-linear layer in different cryptographic ciphers, like Keccak, Xoodoo, and Subterranean.

The mapping $\chi$ can be seen as the application of parallel instances of the mapping $\chi _{\ell }$ to an *n*-bit state partitioned in $\frac{n}{\ell }$ circles of length $\ell$. We refer to such non-linear mapping as a *composite* $\chi$ mapping when $\ell <n$. This is the case for Keccak-$f$, where $\ell =5$, and Xoodoo, where $\ell =3$. Otherwise, we refer to it as a *single-circle* $\chi$ mapping, that is when a single instance of $\chi _{\ell }$ operates on the whole state and $\ell =n$. This is the case for Subterranean, where $\ell =n=257$.

Due to the fact that $\chi _{\ell }$ is translation-invariant [5], it is possible to define it as a local map.

#### Definition 1

The mapping $\chi _{\ell }$ is a transformation of $\mathbb {F}_2^{\ell }$ with local map

1 $$\begin{aligned} y_i = x_{i} + (x_{i+1}+1)\cdot x_{i+2} \, , \end{aligned}$$

where $x$ denotes the input of $\chi _{\ell }$, $y= \chi _{\ell }(x)$ its output, and all indices are taken modulo $\ell$.

An illustration of $\chi _{\ell }$ is given in Fig. .

Equivalently, the mapping $\chi _{\ell }$ can be written as:

2 $$\begin{aligned} \begin{aligned} \chi _{\ell }(x) = x+ (\overline{x} \lll -1)(x\lll -2)&= x+ ((x+(1)^\ell )\lll -1)(x\lll -2) \\&= x+ x\lll -2 + (x\lll -1)(x\lll -2) \\&= x+ x\lll -2 + (x(x\lll -1) \lll -1) \, . \end{aligned} \end{aligned}$$

**Fig. 1:**
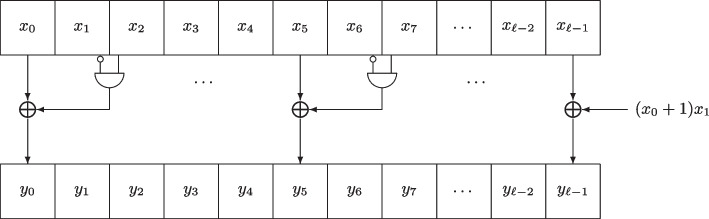
$$\chi _{\ell }$$ transformation

### Simon’s non-linear layer

The Simon cipher is a balanced Feistel network operating on a block of length 2*n*, where $n\in \{32,48,64,96,128\}$. The round function $R_k$ of Simon, illustrated in Fig. , operates on a state consisting of two parts $x$ and $w$, each of length *n*, in the following way:

$$\begin{aligned} \textsf{R}_k(x,w) = (w+g(x)+k,x) \, , \end{aligned}$$

with the function *g* defined as

3 $$\begin{aligned} g(x) = x\lll 2 + (x\lll 1)(x\lll 8) = x\lll 2 + (x(x\lll 7) \lll 1) \, . \end{aligned}$$

**Fig. 2:**
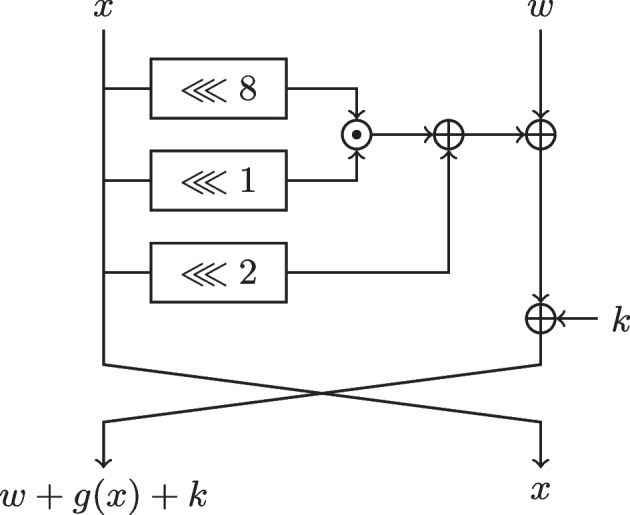
The round function $R_k$ of Simon

### Ascon’s non-linear layer

The Ascon permutation [11] is at the basis of all members of the Ascon cipher suite and is also used in the authenticated encryption scheme Isap.

The non-linear layer of the Ascon permutation is a composite mapping that consists in the parallel application of a 5-bit S-box $\mathcal {S}$, which is depicted in Fig. . The non-linear part of the S-box $\mathcal {S}$ is based on $\chi _5$. It has the same expression of $\chi _5$ but with opposite bit ordering compared to what is used in Keccak. In fact, using the Ascon notation, bit $x_0$ denotes the most significant bit in the 5-tuple of bits. We denote the version of $\chi _{\ell }$ using such opposite ordering as $\widetilde{\chi }_\ell$. More in details, we have:

$$\begin{aligned} \widetilde{\chi }_\ell (x) = x+ (\overline{x} \lll 1)(x\lll 2) = x+ x\lll 2 + (x(x\lll 1) \lll 1) \, . \end{aligned}$$

Therefore, $\mathcal {S}$ can be described as $\widetilde{\chi }_5$ preceded and followed by two linear mappings *A* and *B*, both consisting of 3 bitwise additions:

4 $$\begin{aligned} \mathcal {S}= B\circ \widetilde{\chi }_5 \circ A \, . \end{aligned}$$

**Fig. 3:**
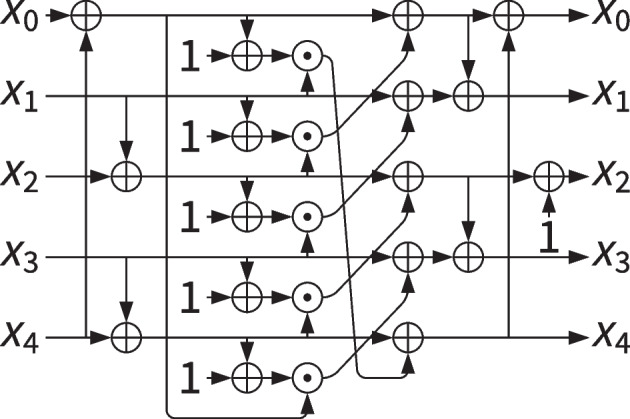
Ascon’s S-box $$\mathcal {S}$$

## Propagation through non-linear layers

In this section, we first recall the main concepts used in differential and linear cryptanalysis. Then, we show that $\chi _{\ell }$ and the non-linear layers of Simon and Ascon are extended-affine equivalent, which allows us to investigate their differential and linear properties in a similar way.

In what follows, we make use of the *extended-affine equivalence* property [3, 17].

### Definition 2

(**Extended-affine equivalence**) Two functions $f:\mathbb {F}_2^{n} \rightarrow \mathbb {F}_2^{m}$ and $f':\mathbb {F}_2^{n} \rightarrow \mathbb {F}_2^{m}$ are extended-affine equivalent (EA-equivalent) if there exist two affine permutations $A:\mathbb {F}_2^{n} \rightarrow \mathbb {F}_2^{n}$, $B:\mathbb {F}_2^{m} \rightarrow \mathbb {F}_2^{m}$ and an affine function $C:\mathbb {F}_2^{n} \rightarrow \mathbb {F}_2^{m}$ such that

$$\begin{aligned} f'(x) = (B \circ f \circ A)(x) + C(x) \, . \end{aligned}$$

### Differential propagation

Let $x,x^{*} \in \mathbb {F}_2^{n}$ be two inputs to a vectorial boolean function $f:\mathbb {F}_2^{n} \rightarrow \mathbb {F}_2^{n}$, and $y, y^{*} \in \mathbb {F}_2^{n}$ be their corresponding outputs, respectively. We call $b= x- x^{*}$ an input difference to *f* and $a= y- y^{*}$ an output difference. A *differential* over *f* consists of a couple $(b,a)$. The number of occurrences of all differentials over a vectorial boolean function $f:\mathbb {F}_2^{n} \rightarrow \mathbb {F}_2^{n}$ are usually arranged in a $2^n$ by $2^n$ array, called the difference distribution table ($\textrm{DDT}$). For a differential $(b,a)$, we have

$$\begin{aligned} \textrm{DDT}(b,a)=|\{x\in \mathbb {F}_2^{n} \mid f(x- b) - f(x) = a\}| \, . \end{aligned}$$

Differential cryptanalysis exploits the probability of differentials to occur.

#### Definition 3

(**Differential Probability** ($\textrm{DP}$)) The differential probability of a differential $(b,a)$ over a function $f:\mathbb {F}_2^{n} \rightarrow \mathbb {F}_2^{n}$ is denoted by $\textrm{DP}(b,a)$ and is defined as

5 $$\begin{aligned} \textrm{DP}(b,a) = \dfrac{\textrm{DDT}(b,a)}{{2^n}} = \dfrac{|\{x\in \mathbb {F}_2^{n} \mid f(x- b) - f(x) = a\}|}{{2^{n}}} \, . \end{aligned}$$

The *differential spectrum* over a function *f* is defined as the number of occurrences of each number in the $\textrm{DDT}$, and is denoted by ${\mathcal D}_f$. Equivalently, the differential spectrum can be defined as the graph representing the number of differentials with a given DP for each DP.

#### Definition 4

(**Differential spectrum**) The differential spectrum of a function $f:\mathbb {F}_2^{n} \rightarrow \mathbb {F}_2^{n}$ is the set

6 $$\begin{aligned} {\mathcal D}_f = \{(p,n)\ \mathrm {s.t.}\ n = \#\{(b,a):\textrm{DP}_f(b,a)=p\}\} \ . \end{aligned}$$

Based on [4, 13], the following lemma holds.

#### Lemma 1

Two EA-equivalent functions have the same differential spectrum.

### Linear propagation

A pair of masks $(u,v)$ with $u$ at the input and $v$ at the output of a vectorial boolean function $f:\mathbb {F}_2^{n} \rightarrow \mathbb {F}_2^{n}$ is called *linear approximation* and satisfies the following relation:

7 $$\begin{aligned} {u}^{\mathsf T} x+ {v}^{\mathsf T} f(x) = 0 \, . \end{aligned}$$

Linear cryptanalysis exploits the *correlation* of linear approximations.

#### Definition 5

(**Correlation**) The correlation of a linear approximation $(u,v)$ over a function $f:\mathbb {F}_2^{n} \rightarrow \mathbb {F}_2^{n}$ is denoted as $\textrm{C}_f(u,v)$ and is defined as

8 $$\begin{aligned} \textrm{C}_f(u,v) = \dfrac{ \{x\in \mathbb {F}_2^{n} \mid \sum _{x}(-1)^{{u}^{\mathsf T} x+ {v}^{\mathsf T} f(x)}\}}{2^{n}} \, . \end{aligned}$$

The correlations of all linear approximations over a vectorial boolean function *f* are usually arranged in a $2^n$ by $2^n$ array, called the linear approximation table ($\textrm{LAT}$). Clearly, correlation can be a positive or negative number. The *extended Walsh spectrum* of a function *f* is defined as the number of occurrences of the absolute value of each number in the $\textrm{LAT}$ and is denoted by ${\mathcal W}_f$.

#### Definition 6

(**Extended Walsh spectrum**) The extended Walsh spectrum of a function $f:\mathbb {F}_2^{n} \rightarrow \mathbb {F}_2^{m}$ is the set

9 $$\begin{aligned} {\mathcal W}_f = \{(c,n)\ \mathrm {s.t.}\ n = \#\{(u,v):\left| \textrm{C}_f(u,v)\right| =c\}\} \ . \end{aligned}$$

Based on [4, 13], the following lemma holds.

#### Lemma 2

Two EA-equivalent functions have the same extended Walsh spectrum.

### Implications for $\chi _{\ell }$, $\textsc {Simon}$ and $\textsc {Ascon}$

The mapping $\chi _{\ell }$ is EA-equivalent to the map $f(x) = x(x\lll -1)$. Using Definition 2, we can take $C(x) = x+ x\lll -2$ and $B(x)=x\lll 1$.

Similarly, we can show that also the map *g* of Simon is EA-equivalent to the map *f*. First, we can observe that *g* is EA-equivalent to the map $g'(x) = x(x\lll 7)$ with $C(x)=x\lll 2$ and $B(x)=x\lll -1$. We can convert the shift by 7 in $g'$ to the shift by -1 in *f*, by taking for *A* a multiplicative shift and for *B* its inverse. This is only possible if the length of the circular state is not a multiple of 7, which is the case for the state sizes used in Simon.

Based on Eq. , the S-box $\mathcal {S}$ of Ascon is EA-equivalent to $\widetilde{\chi }_5$. Similarly to what we observed for $\chi _{\ell }$ and *g*, we can convert $\widetilde{\chi }_5$ into *f*.

It follows that for all these maps, it is sufficient to investigate the differential spectrum and the extended Walsh spectrum of one of them.

## Distribution of differentials over $\chi$

In this section, we report the method presented in [8] to build the differential spectrum of the mapping $\chi$, which is based on counting the number of differentials with a given weight. To this end, we first recall the definition of restriction weight of a differential and the method to compute it over $\chi _{\ell }$ as introduced in [5, Sect. 6.9.1]. Then, we present the method to compute the number of differentials with a given weight $w$ over $\chi$.

### Definition 7

(**Restriction weight**) The restriction weight of a differential $(b,a)$ for a boolean function $f:\mathbb {F}_2^{n}\rightarrow \mathbb {F}_2^n$, denoted by ${\textrm{w}_{\textrm{r}}}(b,a)$, is defined as

$$\begin{aligned} {\textrm{w}_{\textrm{r}}}(b,a) = - \log _2 \textrm{DP}_f(b,a) \, . \end{aligned}$$

In what follows, we use the term *weight* instead of restriction weight when it is clear from the context that we are talking about differentials.

As shown in [5, Sect. 6.9], since the mapping $\chi$ has algebraic degree 2, the weight of any differential $(b,a)$ over $\chi$ is fully determined by $b$. Therefore, we talk about the weight of a difference at the input of $\chi$. When the mapping $\chi$ is composed, the weight of a differential can be computed as the sum of the weights of the differentials over the mappings $\chi _{\ell }$ composing $\chi$. A method to compute the weight over $\chi _{\ell }$ is given in the following proposition.

### Proposition 1

[5, Sect. 6.9] Let $b\in \mathbb {F}_2^{\ell }$ be a difference at the input of $\chi _{\ell }$, and $r$ the number of sub-strings 001 in $b$. The restriction weight of $b$ is given by

10 $$\begin{aligned} {\textrm{w}_{\textrm{r}}}(b) = {\left\{ \begin{array}{ll} \ell - 1 &{} \textrm{if} \ b= (1)^{\ell }, \\ h+ r&{} \mathrm {otherwise.} \end{array}\right. } \end{aligned}$$

### Number of differentials over $\chi$ with a given weight

Clearly, there is only one fully active input difference, and only one fully passive input difference. Therefore, we only consider $h\ne \ell$ and $h\ne 0$.

We denote the number of $\ell$-bit input differences with weight $w$ by ${\mathrm N}(\ell ,w)$. The number of differentials with weight $w$ is $2^{w} {\mathrm N}(\ell ,w)$, as each input difference with weight $w$ leads to $2^{w}$ differences at the output of $\chi _{\ell }$ [5, Sect. 6.9].

With a composite $\chi$ mapping consisting of $q> 1$ applications of $\chi _{\ell }$, we can compute the number of differentials with weight $w$ by $q$-fold convolution using the following recursion formula:

11 $$\begin{aligned} {\mathrm N}(q \times \ell ,w) = \sum _{0 \le x \le w} {\mathrm N}((q-1) \times \ell ,x) {\mathrm N}(\ell ,w-x) \,. \end{aligned}$$

Computing the value of ${\mathrm N}(\ell ,w)$ is easy for small or big values of $w$.

#### Example 1

For $w=\ell -1$, we have ${\mathrm N}(\ell ,\ell -1) = 2\ell +1$ and these are:

- the all-1 string $(1)^{\ell }$;
- $\ell$ translated versions of $0(1)^{\ell -1}$;
- $\ell$ translated versions of $00(1)^{\ell -2}$.

Computing ${\mathrm N}(\ell ,w)$ for an arbitrary $\ell$ and $w$ is not easy. Therefore, we introduce the function ${\mathrm N}_3(\ell ,h,r)$ that computes the number of strings of length $\ell$ with Hamming weight $h$ and containing $r$ 001 sub-strings. Then, the number of $\ell$-bit strings with weight $w$ can be written as

12 $$\begin{aligned} {\mathrm N}(\ell ,w) = \sum _{r= 0}^{\lfloor w/2 \rfloor } {\mathrm N}_3(\ell ,w-r,r) \,. \end{aligned}$$

Here, $r$ is bounded by $\lfloor w/2 \rfloor$ since $w= h+r$, and there cannot be more 001 sub-strings in a string than its Hamming weight, so $r\le h$.

### Computing ${\mathrm N}_3(\ell ,h,r)$

In general, ${\mathrm N}_3(\ell ,h,r)$ is not easy to compute. It is non-zero for a limited range of parameters. In particular, ${\mathrm N}_3(\ell ,h,r) = 0$ if

- $h+2r>\ell$: as any active bit consumes one bit position and every 001 consumes two more bit positions out of $\ell$.
- $r>h$: as there cannot be more 001 sub-strings than active bits.
- $r=0$ and $2h< \ell$: as it is not possible to position less than $\ell /2$ active bits without leaving a gap of two zeroes.

For the other cases, we observe that, since the weight of a string *s* only depends on its Hamming weight and the number of 001 sub-strings in it, deleting a number of specific zeros in *s* will not affect its weight. If a string exhibits a sub-string 101, shortening it by removing the zero in the middle leaves the weight intact as it leaves the Hamming weight and $r$ invariant.

#### Definition 8

(**Hole**) We call the zero in 101 a hole and we denote the number of holes in a string by $x$.

If a string exhibits a sub-string 000, shortening it by removing the leading zero leaves the weight intact as it leaves the Hamming weight and $r$ invariant.

#### Definition 9

(**Pause**) We call the leading zero in 000 a *pause* and denote the number of pauses in a string by $y$.

Now we can define the *minimal difference* as follows.

#### Definition 10

(**Minimal difference**) An input difference of $\chi _{\ell }$ without pauses and holes is called a *minimal difference*. That is, it satisfies $x=0$ and $y=0$.

A minimal difference can be written either as a single 1-run, or as a sequence of 1-runs interleaved with sub-strings 00.

Any string *s* is associated to a unique minimal difference. To prove this, we can observe that the minimal string can be uniquely determined by removing holes and pauses from *s*. First, *s* is transformed by removing holes with the recursive procedure removeHoles in Algorithm 1. This procedure scans *s* and removes the middle 0 from the first 101 sub-string that is encountered. This procedure stops when the string does not contain any more 101 sub-strings. Then, *s* is transformed by removing pauses by applying the recursive procedure removePauses in Algorithm 1. This procedure scans *s* and removes the leading 0 from the first sub-string 000 it encounters. This procedure stops when the string does not contain any 000 sub-strings.
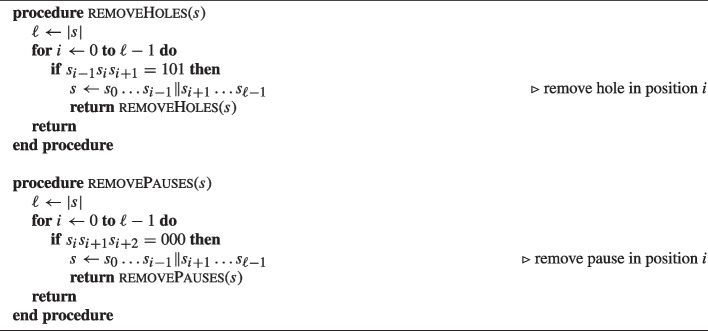


#### Example 2

Let $s = 010010101000001000$. First, holes are removed.

Since $s_4s_5s_6 = 101$, then $s_5$ is removed obtaining $s = 01001101000001000$. Similarly, since $s_{5}s_{6}s_{7} = 101$, then $s_{6}$ is removed obtaining $s = 0100111000001000$.

Since no more 101 sub-strings are contained in *s*, pauses are removed from *s*.

Since $s_7s_8s_9 = 000$, then $s_7$ is removed obtaining $s = 010011100001000$.

Since $s_7s_8s_9 = 000$ again, then $s_7$ is removed obtaining $s = 01001110001000$.

Since $s_7s_8s_9 = 000$ again, then $s_7$ is removed obtaining $s = 0100111001000$.

Then, since $s_{10}s_{11}s_{12} = 000$, then $s_{10}$ is removed obtaining $s = 010011100100$.

Finally, since $s_{10}s_{11}s_{0} = 000$ again, $s_{10}$ is removed obtaining $s = 01001110010$. Since *s* does not contain any 000 sub-string, *s* is a minimal difference.

#### Lemma 3

For a string of length $\ell$, Hamming weight $h$, and containing $r$ 001 sub-strings, $x$ holes and $y$ pauses, the following equivalence holds:

$$\begin{aligned} \ell = h+ y+ x+ 2 r. \end{aligned}$$

#### Proof

The length of a string $\ell$ is the sum of the number of active bits and the number of passive bits. The former is simply the Hamming weight $h$ of the string. The latter is the number of passive bits in each 001 sub-string (i.e., 2) plus the number of passive bits that are removed from the string when reducing it to its minimal difference. Namely, the middle bit of each 101 sub-string and the leading bit of each 000 sub-string. This gives $\ell = h+ y+ x+ 2 r$.

#### Example 3

Let $s = 010010101000001000$ with $\ell = 18$ as in Example 2. The Hamming weight of *s* is $h=5$ and the number of 000 sub-strings is 5 so $y= 5$. The number of 101 sub-strings is 2 so $x= 2$ and finally the number of 001 sub-strings is 3. Then we have $\ell = 5+5+2+2\times 3 = 18$ that is indeed the length of the string *s*.

We denote the number of strings with Hamming weight $h$ and containing $r$ 001 strings, $y$ pauses and $x$ holes by ${\mathrm N}_4(h,r,y,x)$. Lemma 3 implies that

13 $$\begin{aligned} {\mathrm N}_3(\ell ,h,r) = \sum _{x=0}^{\ell -h-2r} {\mathrm N}_4(h,r,\ell - (h+ x+ 2r),x) \,. \end{aligned}$$

### Computing ${\mathrm N}_4(h,r,y,x)$

Since each string can be associated to a unique minimal difference, it follows that the set of strings can be partitioned in classes, where strings in the same class have the same associated minimal difference. Strings in the same class can be built by adding holes and pauses to the corresponding minimal difference in all possible ways. The number of ways to add $x$ holes and $y$ pauses to a minimal difference depends on its Hamming weight and its number of 001 sub-strings, but also on the value of its leading and trailing bits. Namely, it depends on whether the minimal string has a 11 sub-string or a 001 sub-string that spans the boundaries.

#### Example 4

Let $s = 1100111001$ be a minimal difference of length $\ell =10$ and $x=1$. The hole can be placed between any pair of adjacent active bits. But if we consider the pair at indexes $(\ell -1,0)$, then the hole can be placed either at the end of the string or at its beginning. In the former case we obtain the string 11001110010 and in the latter case the string 01100111001.

#### Example 5

Let $s = 011100110$ be a minimal difference of length $\ell =9$ and $y=1$. The unique pause can be placed before any 001 sub-string. If we consider the 001 sub-string at indexes $(\ell -1,0,1)$, then the pause can be placed only between positions $\ell -2$ and $\ell -1$ giving the string 0111001100.

Similarly, let $s = 111001100$ be a minimal difference of length $\ell =9$ and $y=1$. If we consider the 001 sub-string at indexes $(\ell -2,\ell -1,0)$, then the pause can be placed only between positions $\ell -3$ and $\ell -2$ giving the string 1110011000.

#### Example 6

Let $s = 0011111$ be a minimal difference of length $\ell =7$ and $y=1$. If we consider the 001 sub-string at indexes (0, 1, 2), then the pause can be placed between positions $\ell -1$ and 0 in two different ways, giving the strings 00011111 and 00111110. Similarly, for the same string if $y=2$, we can build the strings 000011111, 000111110, 001111100.

We denote the pair $(s_0,s_{\ell -1})$ formed by the leading bit and the trailing bit of a minimal difference *s* by $S$.

Since *s* is minimal, $S=(0,0)$ and $S=(1,0)$ imply that there is a 001 sub-string that crosses the boundaries as in Example 5, while $S=(0,1)$ implies that the string starts with a 001 sub-string as in Example 6. Finally, $S=(1,1)$ implies that there is a 11 sub-string that crosses the boundary as in Example 4.

We denote the number of minimal differences with Hamming weight $h$, $r$ 001 sub-strings and given $S$ by ${\mathrm N}_{min}(h,r,S)$.

It holds that:

14 $$\begin{aligned} {\mathrm N}_4(h,r,y,x) = \sum _{S} \alpha (h,r,x,S) \times \beta (h,r,y,S) \times {\mathrm N}_{min}(h,r,S) \,, \end{aligned}$$

where $\alpha (h,r,x,S)$ denotes the number of ways to add $x$ holes and $\beta (h,r,y,S)$ the number of ways to add $y$ pauses to a minimal state of length $\ell$ with Hamming weight $h$, $r$ 001 sub-strings, and leading and trailing bits specified by $S$.

The factors in the right hand side of (Eq. ) are summarized in Table 1 and we recall how we derived them in the remaining part of this section as presented in [8].

**Table 1:** Combinatorial expressions to compute the number of minimal states of length $\ell$ with Hamming weight $h$, $r$ 001 sub-strings and leading and trailing bits specified by $S$, and the number of ways to add $x$ holes and $y$ pauses to them

$S$	${\mathrm N}_{min}(h,r,S)$	$\alpha (h,r,x,S)$	$\beta (h,r,y,S)$
$S\ne (1,1)$	$$\left( {\begin{array}{c}h-1\\ r-1\end{array}}\right)$$	$$\left( {\begin{array}{c}h-r\\ x\end{array}}\right)$$	$$\left( {\begin{array}{c}y+r\\ y\end{array}}\right) +\left( {\begin{array}{c}y+r-1\\ y\end{array}}\right)$$
$S= (1,1)$	$$\left( {\begin{array}{c}h-1\\ r\end{array}}\right)$$	$$\left( {\begin{array}{c}h-r-1\\ x\end{array}}\right) + 2 \cdot \left( {\begin{array}{c}h-r-1\\ x-1\end{array}}\right)$$	$$\left( {\begin{array}{c}y+r-1\\ y\end{array}}\right)$$

*Case:* $$S\ne (1,1)$$.

#### Proposition 2

The number of minimal differences with Hamming weight $h$ and containing $r$ 001 sub-strings is $$\left( {\begin{array}{c}h-1\\ r-1\end{array}}\right)$$ when $S\ne (1,1)$.

#### Proof

It is the number of ways of putting $r-1$ pairs of passive bits between the $h-1$ pairs of active bits. The position of one 001 sub-string is in fact specified by $S$.

#### Proposition 3

The number of ways to add $x$ holes to a minimal difference with Hamming weight $h$ and containing $r$ 001 sub-strings is $$\left( {\begin{array}{c}h-r\\ x\end{array}}\right)$$ when $S\ne (1,1)$.

#### Proof

It is the number of ways to distribute $x$ holes over $h-r$ positions, where each position can have at most one hole.

Now, we just need to add pauses to the created strings.

#### Proposition 4

The number of ways to add $y$ pauses to a string with Hamming weight $h$ and containing $r$ 001 sub-strings and $x$ holes is $$\left( {\begin{array}{c}y+r\\ y\end{array}}\right) +\left( {\begin{array}{c}y+r-1\\ y\end{array}}\right)$$ when $S\ne (1,1)$.

#### Proof

Here we have two cases:

- $S= (0,1)$: $\beta (h,r,y,S)$ is the number of ways to distribute $y$ pauses over $r$ positions, where there are no restrictions on the number of pauses per position. Since $S= (0,1)$ implies that the minimal difference starts with a 001 sub-string, then the pauses that are put before it can be placed either at the beginning of the string or at its end, as shown in Example 6. It means that there are actually $r+1$ positions to place the $y$ pauses. It follows that $$\beta (h,r,y,S) = \left( {\begin{array}{c}y+r\\ y\end{array}}\right)$$.
- $S\in \{(0,0),(1,0)\}$: $$\beta (h,r,y,S) = \left( {\begin{array}{c}y+r-1\\ y\end{array}}\right)$$, which is the number of ways to distribute $y$ pauses over $r$ positions, where there are no restrictions on the number of pauses per position.

*Case:* $$S= (1,1)$$.

#### Proposition 5

The number of minimal differences with Hamming weight $h$ and containing $r$ 001 sub-strings is $$\left( {\begin{array}{c}h-1\\ r\end{array}}\right)$$ when $S= (1,1)$.

#### Proof

It is the number of ways of putting $r$ pairs of passive bits between the $h-1$ pairs of active bits remaining after excluding the pair at the boundaries.

#### Proposition 6

The number of ways to add $x$ holes to a minimal difference with Hamming weight $h$ and containing $r$ 001 sub-strings is $$\left( {\begin{array}{c}h-r-1\\ x\end{array}}\right) + 2 \cdot \left( {\begin{array}{c}h-r-1\\ x-1\end{array}}\right)$$ when $S= (1,1)$.

#### Proof

$\alpha (h,r,x,S)$ is the number of ways to distribute $x$ holes over $h-r$ positions, where each position can have at most one hole. Since the minimal state starts with a 1 and ends with a 1 then the hole that is placed between these two ones can be placed either at the beginning of the state or at its end, as shown in Example 4. For any other pair of active bits, there is a unique way to put the hole. The total count is thus $$\left( {\begin{array}{c}h-r-1\\ x\end{array}}\right) + 2 \cdot \left( {\begin{array}{c}h-r-1\\ x-1\end{array}}\right)$$, where $$\left( {\begin{array}{c}h-r-1 \\ x-K\end{array}}\right)$$ for $K\in \{0,1\}$ is the number of ways to distribute $x-K$ holes between the $h-r-1$ pairs of ones remaining after excluding the pair across the boundaries.

Now, we just need to add pauses to the created strings.

#### Proposition 7

The number of ways to add $y$ pauses to a string with Hamming weight $h$ and containing $r$ 001 sub-strings and $x$ holes is $$\left( {\begin{array}{c}y+r-1\\ y\end{array}}\right)$$ when $S= (1,1)$.

#### Proof

It is the number of ways to distribute $y$ pauses over $r$ positions, where there are no restrictions on the number of pauses per position.

## Distribution of linear approximations over $\chi$

In this section, we first recall the method to compute correlation weight of a linear approximation over $\chi _{\ell }$ as introduced in [5, Sect. 6.9.1]. Then, we provide a method to compute the number of linear approximations with a given correlation weight $w$ over $\chi$ and present a proof for it.

### Definition 11

(**Correlation weight**) The correlation weight of a linear approximation $(u,v)$ for a boolean function $f:\mathbb {F}_2^{n}\rightarrow \mathbb {F}_2^n$, denoted as ${\textrm{w}_{\textrm{c}}}(u,v)$, is defined as

$$\begin{aligned} {\textrm{w}_{\textrm{c}}}(u,v) = - \log _2 \textrm{C}^{2}(u,v) \, . \end{aligned}$$

In what follows, when it is clear from the context that we are talking about linear approximation over $\chi _{\ell }$, we simply use *weight* instead of correlation weight.

We call 1-runs of odd length *odd-runs* and denote the number of odd-runs in a string by $o$. We also use the term *even-run* for a 1-run of even length. As shown in [5, Sect. 6.9], since the algebraic degree of $\chi _{\ell }$ is two, the weight of any linear approximation over $\chi _{\ell }$ is fully determined by the output mask, and it can be computed using Proposition 8.

### Proposition 8

Given an $\ell$-bit mask $v$ with Hamming weight $h$ and $o$ odd-runs at the output of $\chi _{\ell }$ ($\ell \ge 3)$, its weight is given by:

15 $$\begin{aligned} {\textrm{w}_{\textrm{c}}}(v) = {\left\{ \begin{array}{ll} h+ o- 2 &{} \textrm{if} \ v= (1)^{\ell }, \\ h+ o&{} \mathrm {otherwise.} \end{array}\right. } \end{aligned}$$

### Number of linear approximations over $\chi$ with a given weight

Clearly, there is only one fully active mask ($h=\ell$), and only one fully passive mask ($h=0$). In what follows, we only consider cases where $h\ne \ell$ and $h\ne 0$.

We denote the number of output masks of $\chi _{\ell }$ with weight $w$ by ${\mathrm L}(\ell ,w)$. Then, the number of linear approximations with weight $w$ is simply $2^{w} {\mathrm L}(\ell ,w)$, as each output mask with weight $w$ is correlated to $2^{w}$ masks at the input of $\chi _{\ell }$ [5, Sect. 6.9].

Similar to the differential case, if the $\chi$ mapping is composed by $q> 1$ applications of $\chi _{\ell }$, we efficiently compute the number of linear approximations with weight $w$ by $q$-fold convolution using the following recursion:

16 $$\begin{aligned} {\mathrm L}(q \times \ell ,w) = \sum _{0 \le x \le w} {\mathrm L}((q-1) \times \ell ,x) {\mathrm L}(\ell ,w-x) \,. \end{aligned}$$

To compute ${\mathrm L}(\ell ,w)$, we first provide the following propositions.

#### Proposition 9

Given an output mask with Hamming weight $h$ and containing $o$ odd-runs it holds that $h= o\mod 2$.

#### Proof

Let us denote by 2*e* the total number of active bits in even-runs, and by $2d[i] + 1$ the length of the *i*-th odd-run in an output mask. Then, the Hamming weight of the output mask is:

$$\begin{aligned} h= 2e + \sum _{0}^{o-1}(2d[i] + 1) = 2(e+\sum _{0}^{o-1}d[i]) + o. \end{aligned}$$

Based on Propositions 8 and 9 the following holds.

#### Corollary 1

The correlation weight is always an even number.

Since each 1-run is preceded and followed by a 0-bit, the number of odd-runs is at most equal to the number of passive bits in the output mask. The length of a given output mask is the number of passive bits plus active bits. Hence, $h+ o\le \ell$ and the weight is upper bounded by $\ell$.

#### Corollary 2

For each mask $v$ at the output of $\chi _{\ell }$ the following holds:

$$\begin{aligned} 2\le {\textrm{w}_{\textrm{c}}}(v)\le 2\lfloor \ell /2 \rfloor \end{aligned}$$

Based on Proposition 8, if $v$ has one active bit, then $h+ o= 1 + 1 = 2$; if $v$ has two or more active bits, then ${\textrm{w}_{\textrm{c}}}(v) = h+ o\ge h\ge 2$. On the other hand, the weight is an even number that is upper bounded by $\ell$.

Computing the value of ${\mathrm L}(\ell ,w)$ is easy for small or big weights $w$.

#### Example 7

An output mask with weight $w=2$ can be:

- one of the $\ell$ translated versions of $1(0)^{\ell -1}$; or
- one of the $\ell$ translated versions of $11(0)^{\ell -2}$.

Therefore, in this case, we have

$$\begin{aligned} {\mathrm L}(\ell ,2) = 2 \ell \, . \end{aligned}$$

#### Example 8

Output masks with even length and maximum weight $w=\ell$ have the following form:

- two translated versions of output masks of the form $(1\star )^{\ell /2}$, where starred bits $\star$ can be either 0 or 1, with the exception of the fully active mask.

Therefore, for an even $\ell$, we have

$$\begin{aligned} {\mathrm L}(\ell ,\ell ) = 2\cdot (2^{(\ell /2)}-1) \, . \end{aligned}$$

In general, computing the value of ${\mathrm L}(\ell ,w)$ is not easy for arbitrary $\ell$ and $w$. To simplify this, we introduce a new function ${\mathrm L}_3(\ell ,h,o)$ that computes the number of output masks of length $\ell$ with Hamming weight $h$ and containing $o$ odd-runs. Then, the number of $\ell$-bit masks with weight $w$ for $w\le \ell$ can be written as

17 $$\begin{aligned} {\mathrm L}(\ell ,w) = \sum _{o= 0}^{\lfloor w/2 \rfloor } {\mathrm L}_3(\ell ,w-o,o) \, . \end{aligned}$$

Here, $o$ is bounded by $\lfloor w/2 \rfloor$ since $w= h+o$, and there cannot be more odd-runs than active bits $o\le h$.

### Computing ${\mathrm L}_3(\ell ,h,o)$

In general, ${\mathrm L}_3(\ell ,h,o)$ is not easy to compute. It is non-zero for a limited range of parameters. In particular, ${\mathrm L}_3(\ell ,h,o) = 0$ if

- $$h\ne o\mod 2$$: see Proposition 9.
- $h+o>\ell +1$ and $\ell$ is an odd number: the weight is upper bounded by $\ell$ so, for a non-fully active mask $h+o\le \ell$, and for a fully active mask in this case $h+o= \ell +1$.
- $h+o>\ell$ and $\ell$ is an even number: for a non-fully active mask $h+o\le \ell$, and for a fully active mask in this case $h+ o= \ell$.
- $o>h$: as there cannot be more odd-runs than active bits.

For the other cases, we use the following procedure to calculate ${\mathrm L}_3(\ell ,h,r)$. Since the weight only depends on the Hamming weight and the number of odd-runs, deleting a number of specific zeros will not affect the weight. To compute ${\mathrm L}_3(\ell ,h,o)$, we partition the set into subsets characterized by the specific zeros called *gaps* and *chains*.

If a string exhibits two consecutive zeros, shortening it by removing the leading zero leaves the weight intact since it leaves the Hamming weight and the number of odd-runs invariant. After shortening all two consecutive zeros by deleting the leading zero, if the obtained string starts with an even-run and ends with a single zero, e.g. 11010, deleting the rightmost zero leaves the weight intact.

#### Definition 12

(**Gap**) We call the leading zero in two consecutive zeros a gap. Let *s* be a string that does not contain two consecutive zeros, then if *s* starts with an even-run and ends with 0, we also call the ending zero a gap. We denote the number of gaps in a string by $g$.

We call a string without gaps, a *gap-free* string. In a gap-free string, there are only even-runs and odd-runs that are surrounded by single 0s. Deleting the 0 on the left side of an even-run does not affect the weight since the Hamming weight and the number of odd-runs remain constant.

#### Definition 13

(**Chain**) We call a zero at the left side of an even-run a chain and denote the number of chains by $c$.

#### Definition 14

(**Minimal mask**) An output mask without gaps and chains is called a *minimal mask*.

Any string *s* is associated to a unique minimal mask. To prove this we can observe that the minimal mask can be uniquely determined by removing gaps and chains from *s*. First, *s* is transformed by removing gaps by applying the recursive procedure $\textsc {removeGaps}$ given in Algorithm 2. This procedure scans *s* and removes the leading 0 from the first sub-string 00 it encounters. This procedure stops when the string does not contain any 00 sub-strings. Then, *s* is transformed by removing chains by applying the recursive procedure $\textsc {removeChains}$ given in Algorithm 2. This procedure scans *s* and removes the 0 on the left side of even-runs from the first such 0 that is encountered. This procedure stops when the string does not contain any more 0 on the left side of even-runs.
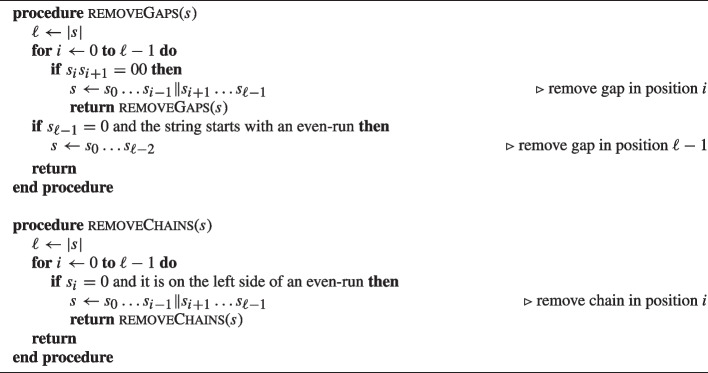


Based on the definition of the minimal mask, the following corollary holds.

#### Corollary 3

Given an output mask that is not fully active or fully passive, its minimal mask can be written either as a single even-run, or as odd-runs preceded and followed by a 0-bit.

#### Example 9

Let $s = 0100101111001$. First, gaps are removed.

Since $s_2s_3 = 00$, then $s_2$ is removed obtaining $s = 010101111001$.

Similarly, since $s_{9}s_{10} = 00$, then $s_{9}$ is removed obtaining $s = 01010111101$.

Since no more 00 sub-strings are contained in *s*, chains are removed in *s*.

Since $s_4 = 0$ and it is located on the left side of an even-run, then $s_4$ is removed obtaining $s = 0101111101$.

Since $s = 0101111101$ does not contain any chain, it is a minimal mask that contains only odd-runs preceded and followed by a 0-bit as stated in Corollary 3.

#### Proposition 10

Given an output mask that is not fully active or fully passive, the number of passive bits in its minimal mask is $o$.

#### Proof

Due to Corollary 3, the number of passive bits in a minimal mask equals the number of odd-runs in it. Given an output mask with $o$ odd-runs, since deleting gaps and chains does not change the number of odd-runs, the number of odd-runs in its minimal mask is $o$.

#### Lemma 4

For a non-fully active mask of length $\ell$, Hamming weight $h$ and containing $g$ gaps, $c$ chains, and $o$ odd-runs we have:

$$\begin{aligned} \ell = h+ c+ g+ o\, . \end{aligned}$$

#### Proof

The length of a mask $\ell$ is the sum of the length of its minimal mask and the number of gaps and chains. The length of the minimal mask is the sum of its Hamming weight and the number of its passive bits $o$ (see Proposition 10).

#### Example 10

Let $s = 0100101111001$ with $\ell = 13$ as in Example 9. The Hamming weight of *s* is $h=7$ and the number of chains is $c=1$. The number of gaps is $g=2$ and finally the number of odd-runs is 3. We have $\ell = 7+1+2+3= 13$ that is indeed the length of the string *s*.

We denote the number of strings of length $\ell$ with Hamming weight $h$ and containing $o$ odd-runs, $g$ gaps and $c$ chains by ${\mathrm L}_4(h,o,g,c)$. Lemma 4 implies that

18 $$\begin{aligned} {\mathrm L}_3(\ell ,h,o) = \sum _{c=0}^{\ell -h-o} {\mathrm L}_4(h,o,\ell - (h+ c+ o),c) \,. \end{aligned}$$

### Computing ${\mathrm L}_4(h,o,g,c)$

Since each string can be associated to a unique minimal mask, it follows that the set of strings can be partitioned in classes, where strings in the same class have the same associated minimal masks. Strings in the same class can be built by adding gaps and chains to the corresponding minimal mask in all possible ways.

We start by generating all the possible minimal masks of length $h+o$ with Hamming weight $h$, $o$ odd-runs and $c=g=0$. Then, we add for each minimal mask $c$ chains to build all possible gap-free strings. Then, we add $g$ gaps to each gap-free string in all possible ways.

Before going on, we need to provide the following definition.

#### Definition 15

(**Even start and odd start masks.**) Without considering the circularity of the string, if a mask starts with 0 or an even-run, we call it $\text {even-start mask}$, otherwise an $\text {odd-start mask}$.

#### Example 11

A mask $s=110101$ is an $\text {even-start mask}$ since (without considering the circularity) it starts with an even-run, namely the 11 sub-string. A mask $s'=101011$ is an $\text {odd-start mask}$ since (without considering the circularity) it starts with an odd-run, namely the 1 sub-string.

Without considering the circularity of the string, the minimal mask associated to an $\text {even-start mask}$ ($\text {odd-start mask}$) should start with 0 or an even-run (odd-run).

#### Proposition 11

The number of minimal masks with Hamming weight $h$, containing $o$ odd-runs, and that form $\text {even-start mask}$s is $$\left( {\begin{array}{c}(h+o)/2\\ o\end{array}}\right)$$, which equals the number of minimal masks with the same $h$ and $o$ that form $\text {odd-start mask}$s.

#### Proof

We start with a string of $o$ odd-runs when all odd-runs have length 1 e.g. for $o= 4$ we have 010101 and 101010. Then we attach 11-strings to each odd-run to get the Hamming weight $h$. The number of 11-strings is $(h-o)/2$. Then, there are two cases:

- $\text {even-start mask}$: then the number of ways to attach $(h-o)/2$ 11-strings to $o+ 1$ positions is $$\left( {\begin{array}{c}((h-o)/2) + (o+ 1) - 1\\ (o+ 1) - 1\end{array}}\right) = \left( {\begin{array}{c}(h+o)/2\\ o\end{array}}\right)$$. We say $o+ 1$ positions since we have $o$ odd-runs, and we can also put 11-strings in the beginning of the string that is also attached to the rightmost odd-run.
- $\text {odd-start mask}$: we have again $$\left( {\begin{array}{c}(h+o)/2\\ o\end{array}}\right)$$ because the number of 11-strings and positions equals the $\text {even-start mask}$ case, since we have $o$ odd-runs and we can also put 11-strings in the end of the string that is also attached to the leftmost odd-run.

Now, we compute the number of gap-free strings by adding $c$ chains to minimal masks.

#### Proposition 12

The number of ways to add $c$ chains to a minimal mask with Hamming weight $h$ and $o$ odd-runs is $$\left( {\begin{array}{c}(h-o)/2\\ c\end{array}}\right)$$ for both cases where the minimal mask forms $\text {even-start mask}$s or $\text {odd-start mask}$s.

#### Proof

The number of ways to put $c$ single chains at the left side of $(h-o)/2$ 11-strings is $$\left( {\begin{array}{c}(h-o)/2\\ c\end{array}}\right)$$.

We only need to add $g$ gaps to gap-free strings to compute ${\mathrm L}_4(h,o,g,c)$.

#### Proposition 13

The number of ways to add $g$ gaps to a gap-free string with Hamming weight $h$ and containing $o$ odd-runs and $c$ chains is $$\left( {\begin{array}{c}c+o+g\\ g\end{array}}\right)$$ for the case of $\text {even-start mask}$, and $$\left( {\begin{array}{c}c+o+g-1\\ g\end{array}}\right)$$ for $\text {odd-start mask}$.

#### Proof

To compute the number of ways to attach $g$ zeros to the passive bits of the gap-free string we consider two cases

- $\text {odd-start mask}$: the number of ways to attach $g$ zeros to $c+o$ passive bits is $$\left( {\begin{array}{c}c+o+g-1\\ c+o-1\end{array}}\right) = \left( {\begin{array}{c}c+o+g-1\\ g\end{array}}\right)$$;
- $\text {even-start mask}$: the number of ways to attach $g$ zeros to $c+o+1$ positions is $$\left( {\begin{array}{c}c+o+1+g-1\\ c+o+1-1\end{array}}\right) = \left( {\begin{array}{c}c+o+g\\ g\end{array}}\right)$$. That is $c+o+1$ positions since we can attach a 0 to either the start or end of the $\text {even-start mask}$.

Using Propositions 11 to 13 allows us to compute ${\mathrm L}_4(h,o,g,c)$ as follows

19 $$\begin{aligned} {\mathrm L}_4(h,o,g,c) = \left( {\begin{array}{c}(h+o)/2\\ o\end{array}}\right) \times \left( {\begin{array}{c}(h-o)/2\\ c\end{array}}\right) \times \left[ \left( {\begin{array}{c}c+o+g\\ g\end{array}}\right) + \left( {\begin{array}{c}c+o+g-1\\ g\end{array}}\right) \right] \,. \end{aligned}$$

We experimentally verified the correctness of Eq. for $\ell \in [3,32]$ by exhaustively generating all $\ell$-bit states, computing their weight using Proposition 8 and checking that the number of states with given weight corresponds to the number obtained by applying our formula.

## Comparing differential and linear properties of $\chi$

In this section, we first investigate the distribution of differentials and linear approximations for some primitives whose non-linear layer is based on $\chi$. For non-linear layers based on composite $\chi$, we provide a comparison between their original definition and *single-circle* $\chi$ mapping, that is when a single $\chi _{\ell }$ operates on the whole *n*-bit state, so that $\ell = n$. Then, we present a comparison between the number of 2-round differential and linear trail cores in Xoodoo that uses $\chi _3$ and Ascon that uses $\mathcal {S}$ that is a variant of $\chi _5$. After that, we investigate the differential properties of $\chi$ for a given output difference, and the linear properties of $\chi$ for a given input mask.

### Differentials and linear approximations

We use the results presented in the previous sections to compute the distribution of differentials and linear approximations in some well known cryptographic primitives, i.e., Subterranean, Simon, Xoodoo, Keccak-$f$[400], and Ascon, whose non-linear layers are based on $\chi$.

For Xoodoo, Keccak-$f$[400], and Ascon, that are based on composite $\chi$, we compare the obtained results with the distribution of differentials and linear approximations of the mapping $\chi _\ell$ when $\ell$ has the state size of such permutations. We underline the fact that we don’t pretend to replace the non-linear layers of such permutations with single-circle $\chi$, as this implies further analysis, which goes beyond the goal of this work. Our goal is to provide a metric for the comparison of these two design approaches, i.e. the usage of composite $\chi$ vs single-circle $\chi$. We chose to use the same state size of the aforementioned permutations for single-circle $\chi$ as an example, but we don’t expect the figures to change significantly when changing the circle size by one or a few more bits.

#### 257-bit state as in Subterranean

We applied the methods introduced in this paper to compute the number of differentials and linear approximations up to a given weight for Subterranean, where $\chi _{\ell }$ operates on the full state of 257 bits. In Fig. a we report the obtained results for $w\le 257$.

**Fig. 4:**
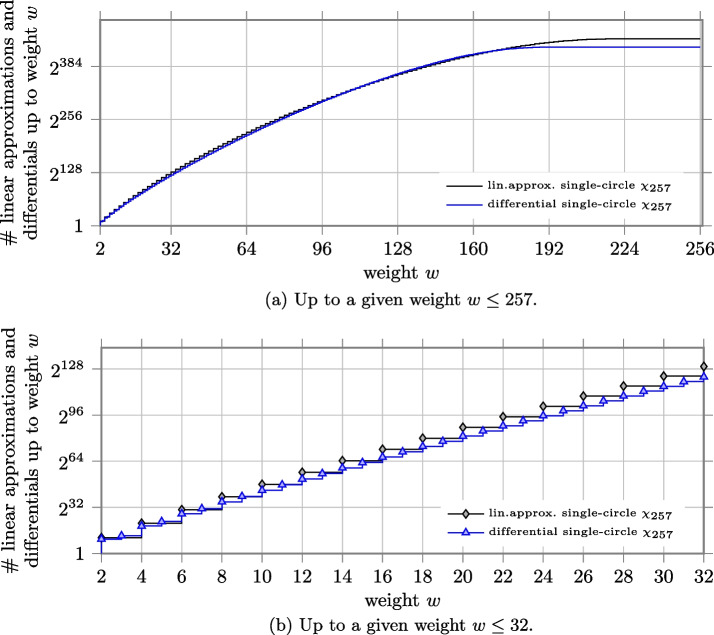
Number of linear approximations and differentials up to a given weight *w* for the case of $\chi _{257}$

Both curves appear flat on the right side because the curves of the number of differentials and linear approximations with given weight are monotonically decreasing after $w = 187$ and $w = 214$ respectively and their contribution becomes negligible. We can notice that the number of linear approximations up to a given (even) weight is in general higher than the number of differentials. To better show this, we zoom on the values up to weight $w\le 32$ in Fig. b.

#### 128-bit state as in Simon

We consider Simon with block length $n=128$ and compute the number of differentials and linear approximations for *g* using $\chi _{128}$. In Fig. a, we report the obtained results for $w\le 128$. Notice that *g* acts on only one block of the state, i.e. *x*. It follows that each differential over *g* corresponds to $2^{128}$ round differentials, since the difference in *w* can be freely chosen.

**Fig. 5:**
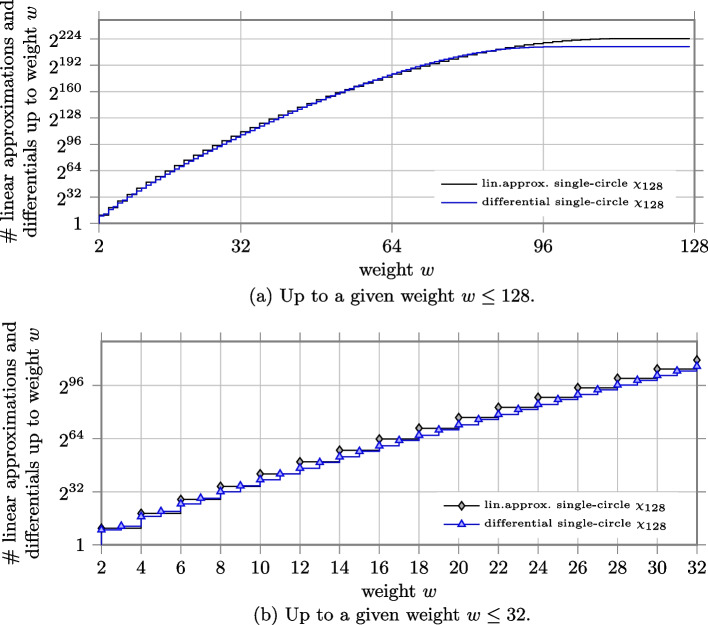
Number of linear approximations and differentials up to a given weight *w* for the case of $\chi _{128}$

As expected, the trend is similar to the case of $\chi _{257}$. Both curves in Fig. a appear flat on the right side because the curves of the number of differentials and linear approximations with given weight are monotonically decreasing after $w = 93$ and $w = 108$ respectively and their contribution becomes negligible. The number of linear approximations up to a given (even) weight is in general higher than the number of differentials. To better show this, we zoom on the values up to weight $w\le 32$ in Fig. b.

#### 384-bit state as in Xoodoo

In the permutation Xoodoo, the state is composed by 384 bits arranged in an array of shape $4\times 3 \times 32$. The non-linear layer is a composite $\chi$ mapping of 128 circles of length $\ell =3$. To compute the number of differentials and linear approximations in Xoodoo, we can use Eqs. 11 and 16. However, we can observe that since both the restriction and correlation weight of any 3-bit circle is 2, then the weight is always even. Therefore, ${\mathrm N}(128 \times 3,w) = {\mathrm L}(128 \times 3,w) =0$ for $w$ odd. For $w$ even,

$$\begin{aligned} {\mathrm N}(128 \times 3,w) = {\mathrm L}(128 \times 3,w) = 7^\frac{w}{2}\cdot {128 \atopwithdelims ()\frac{w}{2}} \end{aligned}$$

that is the number of ways to choose $w/2$ columns among the 128 in the state, multiplied by the possible values of such columns. The number of differentials and linear approximations with restriction weight $w$ is then $2^w\cdot 7^\frac{w}{2}\cdot {128 \atopwithdelims ()\frac{w}{2}}$.

To compute the number of differentials and linear approximations for $\chi _{384}$, we used the formulas introduced in this paper.

We depict in Fig. a the number of differentials and linear approximations with weight smaller than a given weight $w$ for both composite $\chi$ and single-circle $\chi$. Since there are no differentials and linear approximations with weight bigger than 256 in Xoodoo, the histogram for parallel $\chi _{3}$ becomes flat after $w= 256$. The histograms corresponding to the case of single-circle $\chi _{384}$ appear flat toward the end because the curve of the number of differentials and linear approximations with given weight are monotonically decreasing after $w= 288$ and $w= 288$ respectively and their contribution is negligible. We can notice that the number of differentials and linear approximations with high probability is always smaller for the single-circle case. To better show it, we zoom on the values corresponding to weight up to 32 in Fig. b. For instance, the number of differentials and linear approximations with weight up to $w=32$ is $\approx 2^{143.264}$ for composite $\chi$. While for single-circle $\chi$, the number of differentials is $\approx 2^{130.936}$ (i.e., around 5,000 times smaller than the composite case) and the number of linear approximations is $\approx 2^{139.720}$ (i.e., only 11.7 times smaller).

**Fig. 6:**
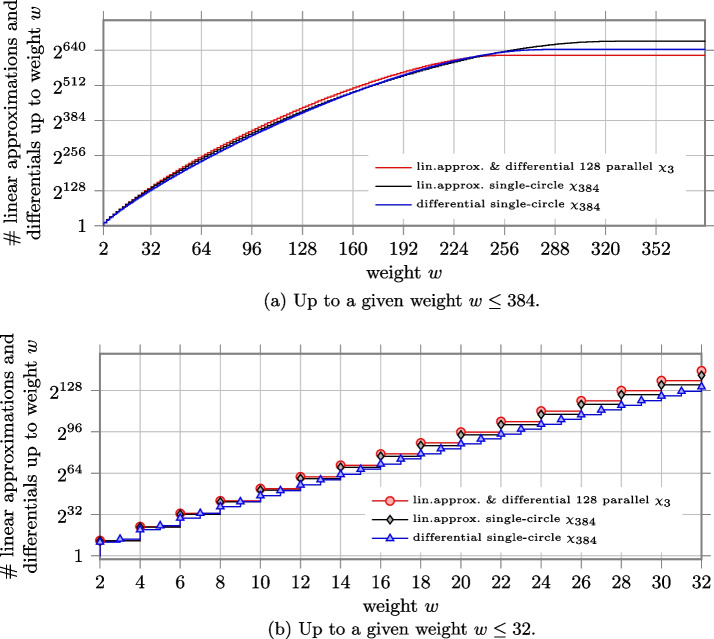
Number of linear approximations and differentials up to a given weight *w* for the case of 128 parallel $\chi _{3}$ as in Xoodoo, and the case of single-circle $\chi _{384}$

Figure a depicts the ratio between the number of differentials up to a given weight for the case of composite $\chi$ over the case of single-circle $\chi _{384}$ and the ratio between the number of linear approximations. Since there are no differentials with odd weight for the case of composite $\chi$, for all $k \in \{0, 1, \ldots 127\}$ the number of differentials with weight smaller than 2*k* and $2k+1$ is the same and the curve presents a zigzag trend. To better show it, we zoom on the values up to weight 32 in Fig. b. We can also observe that for high probabilities, the ratio between the number of linear approximations in the two cases is not so large as the ratio between the number of differentials.

**Fig. 7:**
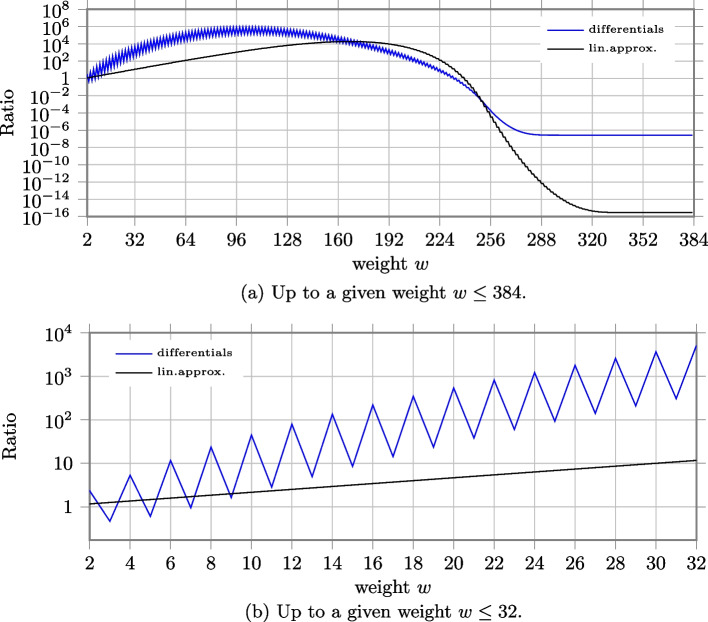
Ratio between the number of linear approximations and differentials up to weight $w$ for the case of 128 parallel $\chi _{3}$ (as in Xoodoo) over the case of single-circle $\chi _{384}$

#### 400-bit state as in Keccak-$f$[400]

In Keccak-$f$[400], the state is organized as an array of $5\times 5\times 16$ bits. The non-linear layer is a composite $\chi$ mapping of 80 circles of length 5, i.e., 80 parallel $\chi _5$.

In Fig. a, we report the number of differentials and linear approximations up to a given weight for composite $\chi$ and single-circle $\chi _{400}$. Compared to the case of $\chi _3$ and $\chi _{384}$, we can observe that now the two curves of differentials and the two curves of linear approximations are closer to each other, with the curves for the case of 80 parallel $\chi _5$ slightly above the others. This can be better noticed in Fig. b, where we focus on the number of differentials and linear approximations up to weight $w\le 32$. For example, the number of differentials with weight up to $w=32$ is $\approx 2^{133.064}$ for composite $\chi$ and $\approx 2^{131.787}$ for single-circle $\chi$. Namely, the latter is around 2.4 times smaller than the former. The number of linear approximations is $\approx 2^{140.764}$ for composite $\chi$ and $\approx 2^{140.719}$ for single-circle $\chi$ and they almost coincide. The ratio between the two cases is depicted in Fig. a for all $w\le 400$, with a focus on $w\le 32$ in Fig. a.

Since the non-linear layer of Ascon is based on $\chi _5$, we have similar results when we compare composite $\chi$ consisting of 64 applications of $\chi _5$ and $\chi _{320}$.

**Fig. 8:**
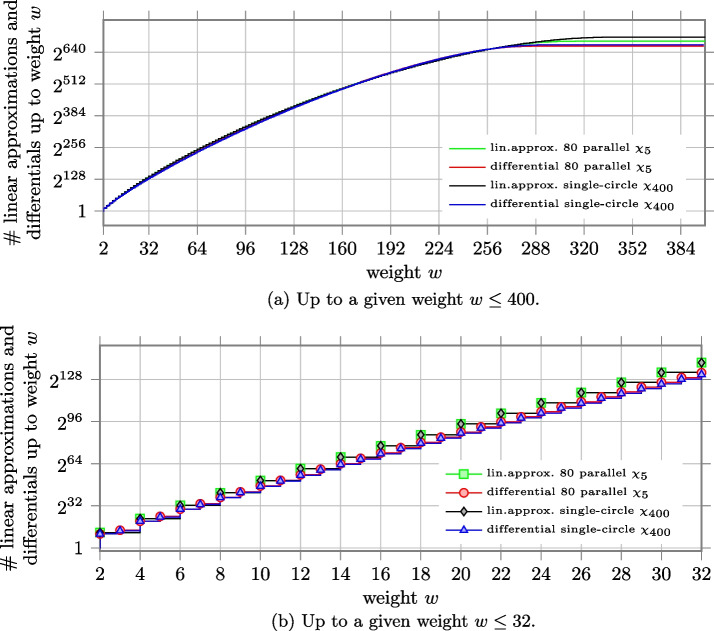
Number of linear approximations and differentials up to a given weight *w* for the case of 80 parallel $\chi _{5}$ as in Keccak-$f$[400], and the case of single-circle $\chi _{400}$

**Fig. 9:**
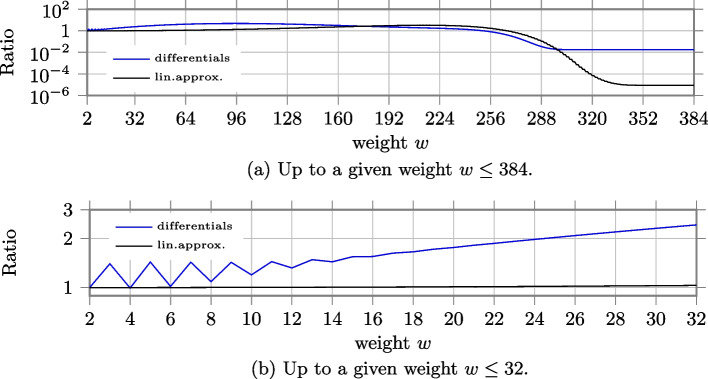
Ratio between the number of linear approximations and differentials up to weight $w$ for the case of 80 parallel $\chi _{5}$ (as in Keccak-$f$[400]) over the case of single-circle $\chi _{400}$

### Number of 2-round differential and linear trail cores

To perform a dedicated trail search as in [15, 16], the first step is to generate all possible 2*-round trail cores* up to a specific weight. Let us denote by $\lambda$ the linear layer of a cryptographic scheme. Then, a 2-round differential trail core consists of a difference $a^{i}$ at the input of $\lambda$, and its corresponding difference $b^{i}$ at the output of $\lambda$, that we represent as $a^{i}\xrightarrow {\lambda } b^{i}$. The weight of a given 2-round differential trail core is computed as $\textrm{w}_{\textrm{rev}}(a^{i}) + {\textrm{w}_{\textrm{r}}}(b^{i})$ where $\textrm{w}_{\textrm{rev}}(a^{i})$ represents the minimum weight over all states $b^{i-1}$ that are compatible with $a^{i}$ through the inverse of the non-linear layer. The weight of a 2-round differential trail core thus lower bounds the weight of all 2-round differential trails with intermediate differences $a^{i}$ and $b^{i}$. A 2-round linear trail core is also defined in a similar way as $v^{i}\xrightarrow {{\lambda }^{\mathsf T}}u^{i}$ with $v^{i}$ the input of ${\lambda }^{\mathsf T}$ and $u^{i}$ its output.

In Fig. we report the number of 2-round linear and differential trail cores with weight $w$ for Xoodoo and Ascon, based on the numbers reported in [9, 14]. We can notice that these results reflect what we observed in Section . In fact, in Fig. we saw that the number of differentials and linear approximations is the same when the scheme uses parallel $\chi _3$. In contrast, as can be seen in Fig. , the number of linear approximations is higher than the number of differentials when parallel $\chi _5$ are used. This results in a kind of balance in the number of linear and differential 2-round trail cores in Xoodoo, and a meaningful difference between these two numbers in Ascon.

**Fig. 10:**
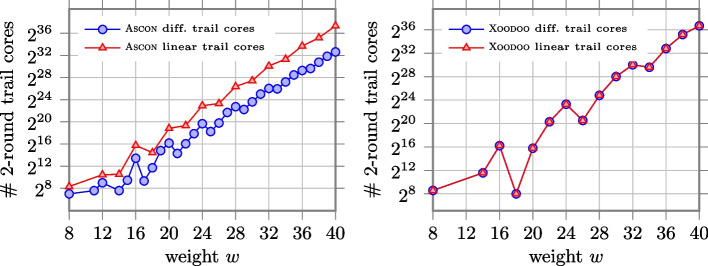
Number of 2-round differential and linear trail cores with a given weight in Ascon (64 parallel $\mathcal {S}$) and Xoodoo (128 parallel $\chi _3$)

### Differential propagation over $\chi$ given an output difference

Given a difference at the input of $\chi _{\ell }$, the way to generate all its output differences is given in [5]. For a given difference at the output of $\chi$, it is possible to build all input differences by brute-force when $\chi$ applies to a short string, but for big strings it is not trivial. To have a better understanding of the differential properties of $\chi$ for a given output difference, we provide a simple example in this section.

We look into the simplest active string, namely a string with a single active bit at the output of $\chi$ and investigate the differential properties of $\chi$ in the backward direction.

A difference $b$ at the input of $\chi$ is called *compatible* with a difference $a$ at the output of $\chi$ if $\textrm{DP}(b,a)>0$. For a single active bit difference $b$, we form a tree of all compatible input differences $a$’s where each node of the tree represents an input difference. The children of a node are generated by adding active bit(s) to that node. Therefore, a specific part of a node remains unchanged while going to the children and descendant nodes. We call the bits in this part *the set bits*. To make our investigation simpler, we assume that the length of the circle is large enough ($\ell \gg 1$), so that there are always at least two passive bits at the right side of the active bit(s) of all nodes in the tree.

Figure represents the tree of the set bits of all input differences that are compatible with a single active bit output difference, up to restriction weight 6. The position of the rightmost active bit of each node is the same as the position of the single active bit of the output and there are always two consecutive zeros at the right side of this active bit. For simplicity, we removed the two zeros at the right side of the set bits in all nodes. For example, the set bits of the first node are (100) but we only depict a single (1).

Each node in Fig. is built by adding

- a (10) to the left side of its parent’s set bits, or
- a (1) to the left side of its parent’s set bits except for the case where its parent node has a pattern (101) on its left.

That can be stated formally as in the following proposition.

#### Proposition 14

Let $a$ be a single active bit output difference and $b$’s be its compatible input differences with at least one active bit at the same position as the single active bit in the output difference followed by two zeros. When generating the tree of all possible $b$’s, adding the following patterns to the left side of a parent node is forbidden:

- two active bits at $b$ with at least two zeros in between e.g. (1001), (10001);
- (1101) pattern at $$b$$.

#### Proof

Since the difference at $a$ should have only one active bit, we need to find compatible $b$’s that add no more active bits to $a$. Based on [7, Propositions 6 and 7], two active bits at $b$ with at least two zeros in between, e.g. (1001), (10001), result in at least two active bits at $a$. Also, the pattern (1101) results in at least two active bits.

**Fig. 11:**
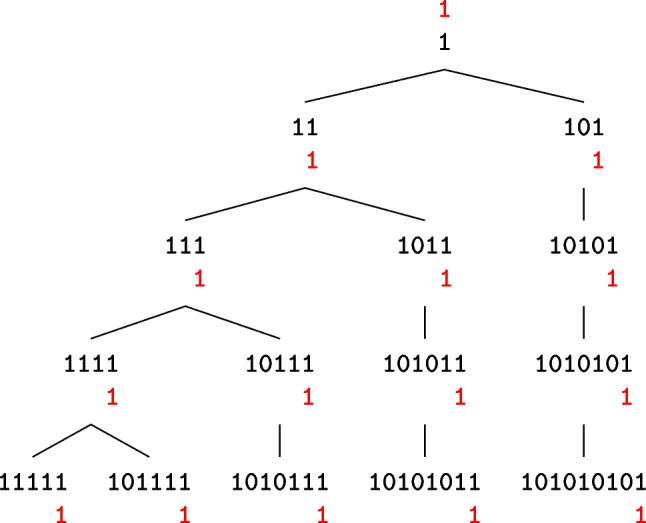
The tree of all input $b$’s (in black) compatible with a single active bit difference $a$ (in red) up to weight 6

The restriction weight of each node in the tree is the restriction weight of its parent node plus 1. That is because adding (1) or (10) to the left side of a node only adds 1 to the Hamming weight, and hence adds 1 to the restriction weight [5]. Hence, all nodes in the same row have the same restriction weight.

Figure represents the number of input differences compatible with a single active bit output difference. We can see that this number grows linearly while increasing the weight.

**Fig. 12:**
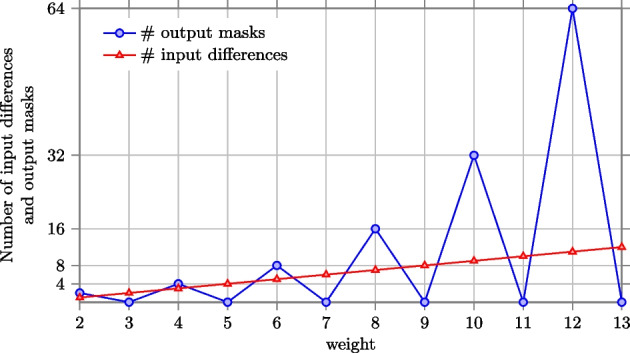
Number of input differences compatible with a single active bit output difference and the number of output masks compatible with a single active bit input mask with given weight

### Linear propagation over $\chi$ given an input mask

Given a mask at the output of $\chi _{\ell }$, the way to generate all its input masks is given in [5]. For a given mask at the input of $\chi$ it is possible to build all output masks by brute-force when $\chi$ applies to short strings, but for long strings it is not trivial. Therefore, we present an example to study the linear properties of $\chi$ for a given input mask with a single active bit.

A linear approximation over $\chi$ has correlation different from 0 if and only if it can be expressed as a sum of product terms plus a constant, as follows:

20 $$\begin{aligned} c_k+\sum (x_i+c_i)\cdot (x_{i+1}+c_{i+1}) \end{aligned}$$

where $c_i$, $c_{i+1}$, and $c_k$ are constants, namely $c_{i},c_{i+1},c_k\in \{0,1\}$.

We say an input mask $u$ is compatible with an output mask $v$ if $\textrm{C}(u,v)\ne 0$. Figure represents the tree of the set bits of all output masks compatible with an input mask with a single active bit, up to correlation weight 6. Here, the position of the leftmost active bit is the same as the single active bit of the input mask and for simplicity, we removed the two zeros at the left side of the set bits in all nodes. Each node in Fig. is generated by adding a mask (10) or (11) to the right side of the set bits of its parent node. Therefore, the following propositions hold.

#### Proposition 15

Let $u$ be an input mask with a single active bit in position *i* and let $v$ be an output mask compatible with $u$. Then, $v$ cannot have two active bits in positions *j*, *k* with two or more zeros in between, with $i\le j<k-2$.

#### Proof

Let us denote a string with a single 1 at index *i* as ${\mathtt e}_{i}\in \mathbb {F}_2^{\ell }$, then $u={\mathtt e}_{i}$. Let *v* have two active bits in positions *j* and *k* with $j+2<k$. Then, *v* is of the form

21 $$\begin{aligned} v=\sum _{\ell<j}{c_\ell {\mathtt e}_\ell }+{\mathtt e}_j+{\mathtt e}_k+\sum _{k<m}{c_m {\mathtt e}_m} \end{aligned}$$

with $$c_{\ell },c_m\in \{0,1\}$$. Then

$$\begin{aligned} \begin{aligned} {u}^{\mathsf T} x+ {v}^{\mathsf T} \chi (x) = x_i + \sum _{\ell<j}{c_\ell (x_\ell + (x_{\ell +1} +1) \cdot x_{\ell +2})} + x_j + (x_{j+1} +1) \cdot x_{j+2}+\\ x_k + (x_{k+1} +1) \cdot x_{k+2}+ \sum _{k<m}{c_m (x_m + (x_{m+1} +1) \cdot x_{m+2})} \, \end{aligned} \end{aligned}$$

Since $j+2<k$, then the term $x_k$ cannot be simplified and the expression above is not of the form of Eq. .

#### Proposition 16

Let $u$ be an input mask with a single active bit in position *i* and $v$’s be the compatible output masks with $v_{i-2}v_{i-1}v_{i}=001$. When generating the tree of all possible $v$’s, adding an active bit proceeded by zero(s), e.g. (01) and (001), to the right side of a parent node is forbidden.

#### Proof

We denote a string with a single 1 at index *i* as ${\mathtt e}_{i}\in \mathbb {F}_2^{\ell }$, then $u={\mathtt e}_{i}$. Based on Proposition 15, having two active bits with at least two zeros in between is not allowed. We prove other cases by induction.

**Base case:**

1. $u= {\mathtt e}_{i}$ is compatible with $v={\mathtt e}_{i}$ because
  $$\begin{aligned} {u}^{\mathsf T} x+ {v}^{\mathsf T} \chi (x) = x_i+x_i + (x_{i+1} +1) \cdot x_{i+2} = (x_{i+1} +1) \cdot x_{i+2} \, \end{aligned}$$
  is a product of two terms $(x_{i+1} +1)$ and $x_{i+2}$, so $\textrm{C}(u,v)\ne 0$.
2. $u= {\mathtt e}_{i}$ is compatible with $v={\mathtt e}_{i}+{\mathtt e}_{i+1}$ because
  $$\begin{aligned} \begin{aligned} {u}^{\mathsf T} x+ {v}^{\mathsf T} \chi (x)&= x_i+x_i + (x_{i+1} +1) \cdot x_{i+2} + x_{i+1} + (x_{i+2} +1) \cdot x_{i+3} \\&=1 + (x_{i+1} +1) \cdot (x_{i+2} +1) + (x_{i+2} +1) \cdot x_{i+3} \end{aligned} \end{aligned}$$
  which is the sum of product terms plus a constant, so $\textrm{C}(u,v)\ne 0$.

**Induction step:**

1. Let us denote a node of the tree that is compatible with $u= {\mathtt e}_{i}$ and has a pattern 10 at its right side by *X*, namely $X_{j-2}X_{j-1} = 10$. Then, the linear approximation over $\chi$ can be represented as $Y + x_{j-1}\cdot x_j+x_j$, where *Y* is a sum of product terms plus a constant, and $x_{j-1}\cdot x_j$ and $x_j$ are the only terms where $x_j$ appears. The reason why we just consider terms with $x_j$ is that these are the only terms that can be affected by adding new bits at positions $\{j, j+1, \dots \}$. Then:
  $u$ is compatible with $v=X+{\mathtt e}_{j}$ because
    $$\begin{aligned} \begin{aligned} {u}^{\mathsf T} x+ {v}^{\mathsf T} \chi (x)&= Y + x_{j-1}\cdot x_j+x_j + x_j + (x_{j+1} +1) \cdot x_{j+2} \\&=Y + x_{j-1}\cdot x_j+ (x_{j+1} +1) \cdot x_{j+2}\, \end{aligned} \end{aligned}$$
    that is a sum of product terms plus a constant, so $\textrm{C}(u,v)\ne 0$;
  $u$ is compatible with $v=X+{\mathtt e}_{j}+{\mathtt e}_{j+1}$ because
    $$\begin{aligned} \begin{aligned} {u}^{\mathsf T} x+ {v}^{\mathsf T} \chi (x)&= Y + x_{j-1}\cdot x_j + x_j + x_j +(x_{j+1} +1) \cdot (x_{j+2} +1) \\&+ (x_{j+2} +1) \cdot x_{j+3} + 1\\&= Y + x_{j-1}\cdot x_j +(x_{j+1} +1) \cdot (x_{j+2} +1) \\&+ (x_{j+2} +1) \cdot x_{j+3} + 1 \end{aligned} \end{aligned}$$
    that is a sum of product terms plus a constant, so $\textrm{C}(u,v)\ne 0$;
  $u$ is **not** compatible with $v=X+{\mathtt e}_{j+1}$ because $v_{j-2}v_{j-1}v_{j}v_{j+1}=1001$ that has two active bits with two zeros in between.
2. Let us denote a node of the tree that is compatible with $u= {\mathtt e}_{j}$ and has a pattern 11 at its right side by *Z*, namely $Z_{j-2}Z_{j-1} = 11$. Then, the linear approximation over $\chi$ is of the form $Y + (x_{j-1} +1)(x_{j} + 1)+(x_{j} + 1) \cdot x_{j+1}$, where *Y* is a sum over products, and $(x_{j-1} +1)(x_{j} + 1)$ and $(x_{j} + 1) \cdot x_{j+1}$ are the only terms where $x_{j}$ and $x_{j+1}$ appear. The reason why we just consider terms with $x_j$ and $x_{j+1}$ is that these are the only terms that can be affected by adding new bits at positions $\{j, j+1, \dots \}$. Then:
  $u$ is compatible with $v=Z+{\mathtt e}_{j}$ because
    $$\begin{aligned} \begin{aligned} {u}^{\mathsf T} x+ {v}^{\mathsf T} \chi (x)&= Y + (x_{j-1} +1)(x_{j} + 1)+(x_{j} + 1) \cdot x_{j+1} \\&+ x_j + (x_{j+1} +1) \cdot x_{j+2} \\&=Y + x_{j-1} \cdot (x_{j} + 1) + (x_{j} + 1) \cdot x_{j+1} + 1 + (x_{j+1} +1) \cdot x_{j+2} \, \end{aligned} \end{aligned}$$
    that is a sum of product terms plus a constant, so $\textrm{C}(u,v)\ne 0$;
  $u$ is compatible with $v=Z+{\mathtt e}_{j}+{\mathtt e}_{j+1}$ because
    $$\begin{aligned} \begin{aligned} {u}^{\mathsf T} x+ {v}^{\mathsf T} \chi (x) =&Y + (x_{j-1} +1)(x_{j} + 1)+(x_{j} + 1) \cdot x_{j+1} \\&+ x_{j} + (x_{j+1} + 1) \cdot (x_{j+2} +1)+ (x_{j+2} + 1) \cdot x_{j+3} +1 \\ =&Y + (x_{j-1} + 1)\cdot (x_{j} + 1) + (x_{j} + 1) \cdot (x_{j+1} + 1) \\ {}&+ (x_{j+1} + 1)\cdot (x_{j+2} + 1) + (x_{j+2} + 1) \cdot x_{j+3} \end{aligned} \end{aligned}$$
    that is a sum of product terms plus a constant, so $\textrm{C}(u,v)\ne 0$;
  $u$ is **not** compatible with $v=Z+{\mathtt e}_{j+1}$ because
    $$\begin{aligned} \begin{aligned} {u}^{\mathsf T} x+ {v}^{\mathsf T} \chi (x) =&Y + (x_{j-1} +1)(x_{j} + 1)+(x_{j} + 1) \cdot x_{j+1}\\&+ x_{j+1} + (x_{j+2} +1) \cdot x_{j+3} \end{aligned} \end{aligned}$$
    and the term $x_{j+1}$ cannot be simplified and the expression above is not of the form of Eq. .

**Fig. 13:**
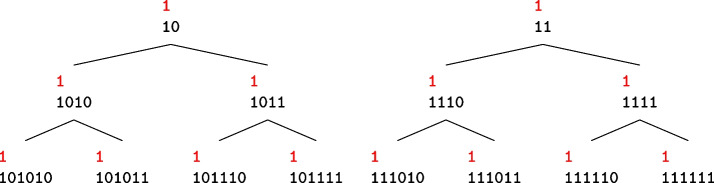
The tree of all output masks $v$’s (in black) compatible with a single active bit mask $u$ (in red) up to weight 6

The correlation weight of each node is equal to the correlation weight of its parent node plus two. This happens because adding (01) to left side of the set bits of each node of the tree in Fig. adds one to the Hamming weight of that node and one to the number of its odd-runs. And, adding (11) to each node only adds two to its Hamming weight. Thus, all nodes in the same row have the same correlation weight.

Figure represents the number of output masks compatible with a single active bit input mask. We can see that this number grows exponentially while increasing the weight, in contrast to the number of input differences which grows linearly.

## Conclusions

We introduced a method to compute the number of linear approximations with a given correlation weight over the non-linear map $\chi _{\ell }$ for any size of $\ell$. Together with the method introduced in [8] to compute the number of differentials with a given restriction weight, this provides a useful tool to evaluate and compare composite $\chi$ mappings and single-circle $\chi$ mappings. Since the non-linear layer of Ascon and Simon are EA-equivalent to $\chi$, our methods can be extended to them.

We used our methods to compare the non-linear layers of ${\textsc {Xoodoo}}{}$ and ${\textsc {Keccak}\text {-}f[400]}$, which use composite $\chi$, with instantiations of single-circle $\chi$. We observed that in the case of ${\textsc {Xoodoo}}{}$, the number of differentials and linear approximations in the case of parallel instances of $\chi _3$ is slightly higher than the single-circle $\chi$. On the contrary, there is no meaningful difference in the case of parallel instances of $\chi _5$ and single-circle $\chi$.

By comparing differentials and linear approximations, we see that the number of linear approximations is the same as differentials in the case of parallel $\chi _3$. Hence, we expect almost the same number of linear and differential trail cores over multiple rounds for the case of parallel $\chi _3$. It confirms the report on the number of 2-round trail cores given in [9]: “We found 2, 983, 444, 980 differential trail cores and 2, 983, 073, 628 linear trail cores. It is worth noticing that these numbers are close to each other within $0.05\%$. This is a consequence of the choice of $\chi _3$ over, for instance, $\chi _5$ as in $\textsc {Keccak}{-}p$”. The authors of [9] also reported on the number of 3-round trail cores, that are 201 in the differential case and 204 in the linear case, that is another confirmation.

Unlike the case of parallel $\chi _3$, the number of linear approximations is bigger than the number of differentials in the case of parallel $\chi _5$ or for longer strings. Therefore, we expect more linear trail cores than differential trail cores over multiple rounds in the case of $\chi$ with a circle length of 5 or greater. The results depicted in Fig. confirm our expectations.
